# Mitochondrial Iron Handling and Lipid Peroxidation as Drivers of Ferroptosis

**DOI:** 10.3390/ijms27052232

**Published:** 2026-02-27

**Authors:** José Luis Bucarey, Mariana Casas, Alejandra Espinosa

**Affiliations:** 1 School of Medicine, Faculty of Medicine, Campus San Felipe, Universidad de Valparaíso, San Felipe 2172972, Chile; jose.bucarey@uv.cl; 2 Center for Studies of Exercise, Metabolism and Cancer (CEMC), Instituto de Ciencias Biomédicas (ICBM), Facultad de Medicina, Universidad de Chile, Santiago 8380494, Chile; mcasas@uchile.cl; 3 Center of Interdisciplinary Biomedical and Engineering Research for Health, Universidad de Valparaíso, San Felipe 2172972, Chile

**Keywords:** mitochondrial dysfunction, ferroptosis, cardiolipin, complex I

## Abstract

Mitochondria are a key organelle in maintaining metabolic homeostasis. It not only generates most of the cell’s energy through oxidative phosphorylation but also acts as a complex sensor of the redox state and oxygen in the cell. This review thoroughly analyzes the interactions among mitochondrial iron metabolism, mitochondrial reactive oxygen species (mtROS), and lipid peroxidation (LPO), the triggering factors of ferroptosis, an iron-dependent form of programmed cell death. We point out research showing that intrinsic mitochondrial machinery, such as iron–sulfur (Fe-S) cluster assembly and heme metabolism, is both an important cofactor and a master regulator. If these processes are disrupted, they can lead to ferroptosis. Unlike views that focus on the cytosol, we explain that the stability of Fe-S clusters in complexes such as aconitase and respiratory Complex I is crucial for preventing electron leakage and excessive mtROS formation. The Fenton reaction and its direct effect on cardiolipin (CL) oxidation in the inner membrane of mitochondria is a central event in cardiometabolic diseases. Its peroxidation and breakdown make the organelle very unstable and lead to cell death though Ca^2+^ overload and a significantly decreased reduced/oxidized glutathione ratio. Additionally, the functions of essential iron transporters and glutathione homeostasis are examined, and their dysregulation is correlated with ferroptosis-associated progression of cardiometabolic and neurodegenerative disorders, such as obesity and Alzheimer’s disease. This review focused on the need to revisit the classic bioenergetic core of the mitochondria as a key player in the pathophysiology of metabolic and neurodegenerative diseases.

## 1. Introduction

In the last decade, mitochondria have been repositioned from a secondary target of oxidative damage to a protagonist, integrating iron metabolism, redox signaling, and bioenergetic control to maintain cell health. Disruption of mitochondrial iron handling has been shown to favor the generation of reactive oxygen species (ROS) and membrane lipid oxidation, thereby creating a permissive environment for the LPO process, and then, ferroptosis [1]. A key hallmark of this type of cellular death is its high regulation, though a machinery that interconnects antioxidant mechanisms, metabolism, and lipid structures integrity, as evidenced by inhibition by antioxidants and iron chelators [2,3]. Indeed, intracellular iron accumulation and LPO metabolites, such as oxidized lipids, are proposed as biomarkers of ferroptosis in disorders such as hepatic ferroptosis and Alzheimer’s disease (AD) [4,5].

In this context, a mechanistic reassessment of mitochondrial iron homeostasis is required to better understand disease pathogenesis and therapeutic responses. In this review, the interactions among iron homeostasis, the antioxidant machinery, and mitochondrial metabolic function will be examined. The relationship between mitochondrial ROS (mtROS) generation and ferroptosis is gaining increasing relevance across metabolic, cardiovascular, and neurodegenerative disorders.

## 2. Cellular and Systemic Iron Management

**Figure 1:**
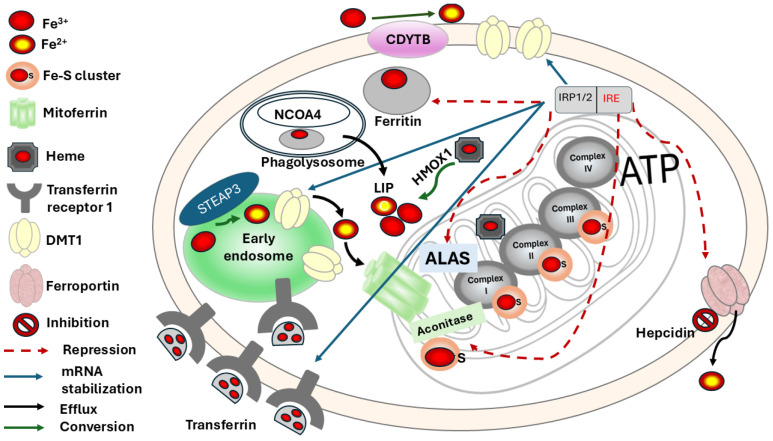
Schematic overview of iron homeostasis under basal conditions. Tf, Transferrin; TFR1, Transferrin Receptor 1; CYB561A3 (DCYTB), Duodenal Cytochrome B (a ferrireductase); LIP, Labile Iron Pool; Ft, Ferritin; NCOA4, Nuclear Receptor Coactivator 4; MFRN1, Mitoferrin-1; ALAS, Aminolevulinic Acid Synthase; Fe-S, Iron–Sulfur cluster; I, II, III, IV, Mitochondrial respiratory complexes; ATP, Adenosine Triphosphate; HMOX1, Heme Oxygenase 1; IRP1/2, Iron Regulatory Proteins 1 and 2; IRE, Iron Responsive Element.

Cellular management of iron includes proteins involved in uptake, storage, redox modifications, importers and exporters, Fe clusters, and heme. Iron enters the body through the diet and is then absorbed by enterocytes in the duodenum and the proximal jejunum, where 1–2 mg of the 10–15 mg provided by a normal diet are reabsorbed. Only between 20 and 40% of the heme group from myoglobin and hemoglobin provided by the diet is available for intestinal absorption. Some vegetables and cereals provide non-heme iron, of which only 10–20% is reabsorbed [6]. Iron is a metal that exists in the divalent ferrous (Fe^2+^) and trivalent ferric (Fe^3+^) redox states, which allows for electron transfer in cellular processes [7]. To maintain iron concentration within the optimal physiological range, regulation must be maintained at both the cellular and systemic levels. Iron regulation inside the cells should be described in Figure 1. During intestinal absorption, the divalent metal transporter 1 (DMT1), expressed in enterocytes, imports dietary iron, but it is also expressed in the membranes of other cells that require non-heme iron [8]. The protein ferroportin 1 (FPN1), encoded by the *SLC40A1* gene, is in the basal membrane of the enterocyte, Kupffer cell in the liver, in the placenta, and in macrophages, and exports iron to the circulation. In fact, an autosomal recessive hereditary disorder called ferroportin disease causes iron overload in these patients [9]. Then, plasma transferrin (Tf), an iron-transport protein, binds and transports iron to tissues such as hematopoietic and hepatic tissues, where it is used or stored [10]. To enter the cells, iron binds to a specific receptor called transferrin receptor 1 (TfR1). Once bound to this receptor, iron forms a complex that is endocytosed [11]. The acidification of the endosome facilitates the release of iron from Tf. This ferric iron (Fe^3+^) is then reduced to ferrous iron (Fe^2+^) by the metalloreductase CYB561A3 (DCYTB) and transported across the endosomal membrane into the labile iron pool (LIP). Iron storage occurs when there is an excess of the metal, which is primarily stored in ferritin within the mononuclear phagocyte system. Specifically, it is produced by Kupffer cells in the liver, red pulp macrophages in the spleen, and alveolar macrophages in the lungs. Also, a high expression is observed in erythroid precursors within the bone marrow. Additionally, ferritin is synthesized by distinct populations of neurons and glial cells in the central nervous system [12]. The release of iron from ferritin occurs via a process called ferritinophagy, mediated by the cargo receptor Nuclear receptor coactivator 4 (NCOA4), which delivers ferritin to the phagolysosome for degradation [13]. Iron is imported into the mitochondria via mitoferrin to be incorporated into heme and hemoprotein. Heme synthesis, a process initiated by the rate-limiting enzyme Aminolevulinic Acid Synthase (ALAS), involves the formation of iron–sulfur (Fe-S) clusters, as well as the cytosolic next step catalyzed by cytosolic enzyme aminolevulinic acid dehydratase (ALAD) [14]. The Fe-S cluster assembly machinery supports the function of numerous proteins, including mitochondrial Complex I, II, and III of the electron transport chain (ETC), enabling ATP production via oxidative phosphorylation (OXPHOS). In the cytosol, Fe-S clusters are also incorporated into aconitase, converting it into its enzymatic form.

The liver-derived peptide hormone hepcidin is a negative regulator of iron homeostasis, binding to the iron exporter ferroportin, leading to its internalization and degradation [15]. Regulation of iron homeostasis inside the cell is commanded by a transcriptional and degradative pathway, such as heme oxygenase (HMOX-1), which degrades heme, releasing Fe^2+^ to LIP. Iron regulatory proteins 1 and 2 (IRP1/2) are post-transcriptional regulators that, under low iron conditions, bind to the iron-responsive element (IRE) present in the 5′ or 3′ untranslated regions (UTR) of their respective mRNAs, as shown in Figure 1. Proteins such as TFR1 and ferritin or DMT1 are stabilized after the union of IRP1/2 to IRE, and others, such as ferritin or ALAS, are repressed from translation.

On the contrary, when there is an adequate concentration of iron, the increase in iron levels results in the loss of IRP affinity for IRE, leading to an increase in the translation of mRNA and the production of ferritin and a decrease in the iron importer proteins [7].

## 3. Healthy Mitochondrial Function Is Crucial for a Metabolically Healthy Cell

Cellular metabolism depends on a healthy mitochondrial function. Mitochondria are dynamic metabolic centers that primarily produce energy for the cell via OXPHOS. The generation of ATP via the ETC and the maintenance of its membrane potential are hallmarks of healthy mitochondrial function. The ETC is fed by molecules derived from the Krebs cycle, such as NADH and FADH2, which donate electrons to complex I and II, respectively, located in the inner mitochondrial membrane. Structurally, the ETC is a macromolecular supercomplex (respirasome) that organizes its components within the inner mitochondrial membrane to enhance electron flow and reduce ROS production [16].

The Krebs cycle is not only an essential core of metabolism but also contributes to the cell’s oxygen sensor. Prolyl-hydroxylases (PHDs) are oxygen sensor-regulated by 2-oxoglutarate, succinate, fumarate, and isocitrate, all of which are Krebs ’ cycle intermediates, in order to modulate gene expression and metabolism [17,18]. One of the most characteristic examples is hypoxic-induced factor alpha (HIFα), in which a proline residue can be hydroxylated by oxygen or α-ketoglutarate [19,20]. This signal induces its degradation, which is why HIFα cannot bind to HIFβ to form the active transcription factor dimer [21]. This is a clear connection between metabolically healthy mitochondria and the oxygen sensor capacity.

Mitochondria are also an active part of the cell’s redox homeostasis. ROS generation is part of physiological signal transduction, acting as second messengers, and mtROS are generated during respiration [22]. Both superoxide anion (O_2_•^−^) and hydrogen peroxide (H_2_O_2_) originate from complexes I, II, and III of the respiratory chain, at specific sites. Mitochondria are oriented in specific subcellular locations to conserve energy and minimize mtROS production, thereby reducing potential damage [23]. When an energetic substrate is offered to the cell, electron flux through the ETC increases, raising mitochondrial membrane potential and producing mtROS from complex I. This overproduction of mtROS inhibits key enzymes and proteins, such as pyruvate dehydrogenase kinase [24]. In this way, pyruvate would not be converted to acetyl-CoA, or mtROS would modify the redox status of uncoupling proteins (UCPs), providing protection against dysregulated hyperpolarization.

Healthy mitochondria have a powerful antioxidant machinery; the glutathione system is present in the mitochondrial matrix as a first line of defense against excess mtROS, which can reach concentrations higher than in the cytosol. Reduced glutathione (GSH) is formed continuously from the reduction of oxidized glutathione (GSSG) and is incorporated by its mitochondrial transport SLC25A39 [25]. Both molecules are tripeptides composed of γ-Glu-Cys-Gly; therefore, the cysteine concentration within the matrix is a crucial factor in maintaining adequate GSH levels. This system is powered by glutathione reductase, a NADPH-dependent enzyme that reduces one GSSG to two GSH. A healthy mitochondrion requires sufficient NADPH in its matrix to maintain a GSH/GSSG ratio > 10:1 [26]. Another clue enzymes are ϒ-Glutamylcysteine synthetase and Glutathione synthetase, both present in the mitochondrial matrix, and both are involved in the de novo synthesis of glutathione. Glutathione peroxidase 4 (GPX4) is present in both the matrix and the inner membrane of the mitochondria. Its activity depends on selenium to reduce H_2_O_2_ and peroxidized lipids [27].

Both the respiratory chain and OXPHOS are iron-dependent processes because complexes I, II, and III require Fe-S clusters as cofactors for their function, and cytochrome c and Complex IV contain heme groups [28]. Iron–sulfur clusters vary from simple [1FeS] rubredoxins to complex [8Fe–7S] nitrogenases. Common forms include [2Fe–2S], [3Fe–4S], and [4Fe–4S], typically cysteine-ligated, with histidine ligation in Rieske proteins; the latter are present in the complex III of the ETC [28].

Mitochondrial metabolism works together with heme synthesis. Careful iron management by mitochondria is essential for their own survival and for the metabolic homeostasis of the individual.

## 4. Mitochondrial Iron Handling

### 4.1. Fe-S Clusters

Several cellular functions, such as electron transfer in metabolism, depend on metalloproteinases that require Fe-S clusters for activity. The binding of the Fe-S cluster to proteins is through a cysteine or a histidine. Healthy mitochondria have this protein with a Fe-S cluster, which regulates metabolic pathways and serves as a redox sensor. Table 1 shows the Fe-S cluster relevant for mitochondrial metabolism.

As was mentioned before, mitochondria are a central hub for cellular redox homeostasis, requiring several complexes with Fe-S cluster, which are directly involved in the controlled, stepwise production of mtROS. The efficiency of ETC depends on the proper assembly of Fe-S clusters, as without them, electron leakage could increase, leading to uncontrolled mtROS generation. If the ETC becomes over-reduced or if a Fe-S cluster within a protein is damaged or incorrectly assembled, electrons can prematurely escape molecular oxygen, generating superoxide. Fe-S can be oxidized or disassembled; these modifications affect metabolic enzymes, such as aconitase and transcription factors (Table 1). Fe-S clusters are crucial for mitochondrial homeostasis, serving as redox sensors, minimizing mtROS generation, and acting as sentinels of the cell’s energetic metabolic status, even intracellular pH changes [29,30].

**Table 1:** Fe-S clusters relevant for mitochondrial metabolism.

Cluster	Mitochondrial Location	Metabolic Function	References
[2Fe-2S]	Complex I (N1a, N1b clusters)	Electron Transfer: Mediates a single electron jumps within the ETC and key redox-active components.	[31,32]
	Complex II	Involved in the biosynthesis of steroids, heme and lipoyl cofactors.	[33,34]
	Complex III (Rieske protein)	The Rieske cluster moves physically to facilitate electron transfer from ubiquinol to cytochrome c. Tune the activity of monooxygenase TsaM.	[35,36]
	Mitochondrial matrix	Regulator: Molecular sensors (Cysteine Desulfurase 1, NFS1; Iron–Sulfur Cluster Scaffold Protein, ISCU, and Glutaredoxin-related protein 5, GLRX5) incorporated into SLC25A39 (GSH transport)	[25,37]
[3Fe-4S]	Complex II (terminal cluster)	Electron Transfer: Aligned near the quinone binding site in Complex II.	[38]
	Mitochondrial Aconitase (inactive form)	Redox Sensing: The inactive aconitase contains this cluster; it transitions to the [4Fe-4S] upon acquiring a labile iron atom.	[39,40]
[4Fe-4S]	Complex I (N2, N3, N4, N5, N6a, N6b clusters)	Enzyme Catalysis: catalyzes the conversion of citrate to isocitrate via aconitase in the Krebs cycle	[41,42]
	Complex II (middle cluster Mitochondrial)	Oxygen Sensing: The N2 cluster in subunit Ndufs2 acts as a redox-sensitive oxygen sensor.	[43]
	Aconitase (active form)	Electron Tunneling: Forms a tunneling chain over 95 Å in the Complex I to drive proton pumping	[44,45]
Cluster N2	Complex I (Subunits NDUFS7/NDUFS2)	Terminal Sink: Acts as the high-potential electron sink that reduces ubiquinone to ubiquinol	[46]
		Homeostatic oxygen-sensing system (HOSS) Regulation: Vital for homeostatic oxygen-sensing systems in pulmonary arteries and the carotid body	[47,48]
Rieske	Complex III (Iron Sulfur Protein)	Bifurcated Electron Flow: Participates in the “high potential pathway” of the Q-cycle, transferring electrons to cytochrome c1	[49,50]

### 4.2. Heme Group

On the other hand, mitochondria play a pivotal role in heme synthesis and its regulation. The pro-oxidant nature of heme could generate oxidative intracellular damage. Its accumulation can disrupt redox balance, as elevated iron and ROS levels can shift the cell’s state from a protective to a pro-death state [51]. The heme group is part of the structure of a large number of enzymes, in and out of the mitochondria. It has been proposed that the enzymes of heme biosynthesis exist as a metabolon, a protein complex formed by interacting enzymes that provide a scaffold for interactions with other pathways [52]. The heme group is a metalloporphyrin composed of a tetrapyrrolic porphyrin ring that coordinates a central iron atom, an essential cofactor for its biochemical function. The canonical heme biosynthetic pathway comprises eight sequentially acting enzymes and is initiated in the mitochondrial matrix by the condensation of succinyl-CoA and glycine to form ALAS, a reaction catalyzed by the pyridoxal-5′-phosphate–dependent enzyme 5-aminolevulinate synthase [53]. Then, ALAS is transported to the cytosol, where it is converted to porphobilinogen and subsequently processed into coproporphyrinogen III. This intermediate re-enters the mitochondria for oxidation and final iron insertion by ferrochelatase, which inserts ferrous iron into protoporphyrin IX, completing heme synthesis [54].

Mitochondria regulate heme synthesis through different proteins, such as the complex IV assembly cofactor heme A:farnesyltransferase (COX-10), whose nephron-specific loss leads to mitochondrial dysfunction, severe kidney failure, and premature death, linking impaired mitochondrial respiration to innate immune activation through interferon signaling in renal epithelial cells [55]. In the same context, healthy mitochondria can respond to hypoxia through miR-210, which diminishes cellular heme concentrations and the functionality of mitochondrial and cytosolic heme-dependent proteins by regulating ferrochelatase, the terminal enzyme in heme biosynthesis [56].

Heme is part of the structural conformation of a member of the ETC; complex II (succinate dehydrogenase) requires a heme group only for structural stabilization, because the electrons from FADH_2_ flow directly through the [Fe-S] centers to ubiquinone, bypassing this heme [57]. The two b-type hemes (b_l_ and b_h_) in the cytochrome b of Complex III are integral to the Q-cycle process [58]. Their unique redox potential establishes a pathway that reverses direction, from the electron pair to ubiquinol. This division allocates one electron to the Rieske cluster and cytochrome c_1_, while the other returns through the b-hemes to decrease an additional quinone [59]. This distinctive electron bifurcation is the essential process that allows the complex to translocate protons and save energy. Therefore, without the heme group in complex III, electron transfer could not occur.

The Complex IV or cytochrome c oxidase has a specialized structure with metallic cores, and has two heme groups: cytochrome α and cytochrome α3. Cytochrome α carries a formyl group (-CHO), which serves as a transient electron-transfer site in redox homeostasis. A binuclear core with copper (Cu-A and Cu-B) is part of the structure. The last ones are essential for oxygen reduction; Cu-A is an initial electron donor, and cytochrome α3 is associated with the Cu-B center, where molecular oxygen is bound and reduced to water [60]. Both Fe-S clusters and heme groups are essential chemical structures for redox homeostasis, balanced mtROS generation, and mitochondrial metabolic function.

## 5. Lipoperoxidation Process in Mitochondrial Dysfunction

Several metabolic disturbances characterized by mitochondrial dysfunction include cardiometabolic disease, such as clinical obesity, type 2 diabetes, and metabolically associated steatotic liver disease (MASLD) [61,62,63]. Mitochondrial dysfunction is a set of structural, functional, and molecular defects in mitochondria. Among the main hallmarks are reductions in respiratory capacity and OXPHOS, an increase in oxidative stress that damages membrane lipids, proteins, and mtDNA, alterations in mitochondrial dynamics (disequilibrium between fusion and fission), disruption of energetic metabolism, mtDNA mutations, and activation of apoptosis [64].

The lipoperoxidation process and mitochondrial dysfunction are a vicious cycle of bioregulatory impairment and oxidative stress. Mitochondrial membranes are damaged, amplifying this damage throughout the organelles. Lipoperoxidation occurs when ROS react with polyunsaturated fatty acids (PUFAs) to generate lipid peroxides, a process associated with degenerative chronic diseases with an inflammatory component [65]. LPO occurs in three phases: initiation, propagation, and termination. Initiation could be triggered by both the hydroxyl radical (OH•) or the hydroperoxyl radical (OOH•), ROS-derived by the Fenton reaction or by peroxynitrite (ONOO-), RNS-derived. This step implies the abstraction of a hydrogen atom from the allylic carbon to form a lipid radical (L•). The second phase consists of the reaction of (L•) with oxygen to form a peroxyl radical (LOO•), which can then abstract another allylic carbon to form a new (L•) and a lipid peroxide (LOOH), thereby propagating the chain reaction. The termination phase is characterized by the accumulation of high amounts of peroxyl radical. When they react and form a new bond, the radical is eliminated. Then, the Fenton reaction is a key step in which iron initiates lipid peroxidation [66]. Among the reactive lipid species generated by lipid peroxidation are 4-hydroxy-2-nonenal (4-HNE) and malondialdehyde (MDA), both of which are used as biomarkers of lipid peroxidation [67]. Lipoperoxidation in mitochondrial membranes is initiated for the generation of superoxide anion (O_2_•^−^) in the ETC. The inner membrane has a microdomain rich in CL, whose structure has linoleic acid (C18:2), and is negatively charged, generating a local environment with low pH relative to the interfacial membrane-matrix. Low pH induces the conversion of O_2_•^−^ to hydroperoxyl radical (HO_2_•), highly reactive with PUFA, above all CL and phosphatidyl ethanolamine [68]. This reaction triggers the isoprostane pathway of lipoperoxidation, generating a racemic mixture of highly toxic isoprostanes and isoketals, or linoleic acid hydroperoxides. These oxidative products irreversibly alter the structural and functional integrity of respiratory supercomplexes and ATP synthase, compromising oxidative phosphorylation [69].

## 6. Lipoperoxidation as a Driver of Ferroptosis

Lipid peroxides themselves can induce mitochondrial damage by impairing membrane integrity, disrupting respiratory complex function, opening the mitochondrial permeability transition pore (mPTP), and inducing the release of cytochrome c and mitochondrial swelling, leading to cell death [70]. LPO in the mitochondrial inner membrane alters the integrity and function of calcium transporters, such as Na^+^/Ca^2+^ (mNCE). Consequently, Ca^2+^ is trapped inside, and depolarization further exacerbates the problem, triggering Ca^2+^ influx through the mitochondrial calcium uniporter (MCU) [71]. This defective Ca^2+^ homeostasis, a hallmark of mitochondrial dysfunction, could be prevented by mito-ROS scavenging, as reported in ventricular myocytes, restoring Ca^2+^ homeostasis [72].

Given the highly regulated mitochondrial iron management, iron can be dangerous only when mitochondria are not healthy. Free Fe^2+^ could react with H_2_O_2_ through the Fenton reaction that generates hydroxyl radical (OH•) [66], and lipids such as CL are susceptible to this type of radical species. When a radical attack occurs in CL, lipid peroxidation is initiated in the inner mitochondrial membrane.

**Figure 2:**
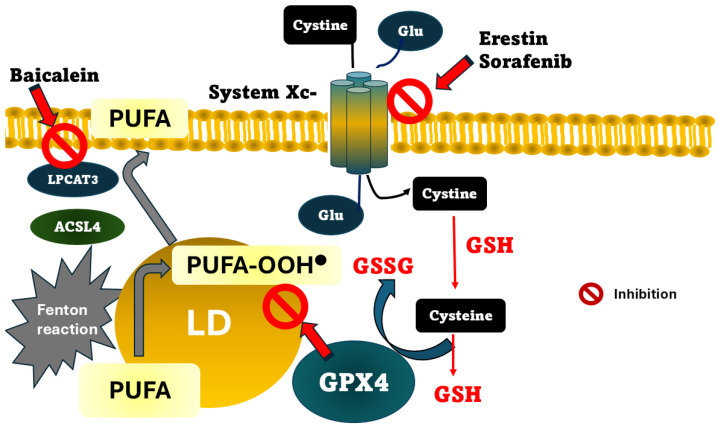
Mechanisms by which ferroptosis could be produced. The pathways for eliminating LPO products (represented as PUFA-OOH^•^) include system Xc, GSH/GPX4 axis, and Fenton reaction. Cystine uptake is produced by system Xc-, and is catalyzed to GSH/GSS recycling. GPX4 converts GSH to GSSH, reducing LPO and inhibiting ferroptosis. Main inhibitors are shown. PUFA, polyunsaturated fatty acids; LD, lipid droplet; ACSL4, acyl-CoA synthetase long-chain family member; GR, glutathione reductase; LPCAT3, lysophosphatidylcholine acyltransferase 3; GPX4, glutathione peroxidase 4.

Ferroptosis is an iron-dependent mechanism of cell death. This concept was introduced in 2012 by Hirschhorn and Stockwell as a cellular death phenotype morphologically distinct from autophagic, apoptotic, or necrotic cell death [73]. The concept has been enriched with evidence related to the highly regulated process, dependent on GSH homeostasis, in which key enzymes, such as GPX4 and cysteine supply, are crucial for preventing it [74]. Currently, three mechanisms by which ferroptosis can occur have been proposed. One is the inhibition of the glutamate/cystine antiporter system Xc− (Figure 2). This transporter imports cystine into the cell, which is used for GSH synthesis. This importer is the rate-limiting substrate for GSH synthesis. Erastin and sorafenib are drugs that inhibit the system Xc− activity, producing the accumulation of peroxidized lipids, and are an effective tool for inducing in vitro ferroptosis [75]. Given that GSH is considered one of the cell’s essential components in counteracting LPO, acting by donating a hydrogen atom to yield a nonradical product, lipid peroxides can also be reduced through a mechanism involving GPX4, with GSH being utilized as a cofactor [76]. Consequently, the second mechanism underlying increased lipid peroxidation is the loss or depletion of GPX4 activity. GPX4 is a selenoprotein containing the rare amino acid selenocysteine at its active site, which requires post-translational modification, such as palmitoylation, to be stable [77]. Following GPX4 action, lipid peroxides are converted to their corresponding alcohols or aldehydes, thereby halting the propagation phase of lipid peroxidation. Consequently, GPX4 is widely recognized as a critical regulator of ferroptosis. Its primary antioxidant function is the reduction of phospholipid hydroperoxides within cellular membranes. Ferroptosis is induced by genetic or pharmacological inhibition of GPX4, leading to elevated lipid peroxidation products that are considered to favor the progression of MASLD. One example of such pharmacological inhibition is RSL3, a ferroptosis inducer whose mechanism of action involves the specific inhibition of GPX4 and has been proposed to target colon cancer [78]. However, there is another complementary cellular death protective pathway, mediated by Ferroptosis Suppressor Protein 1 (FSP1), a key protein that inhibits ferroptosis by reducing coenzyme Q10 to ubiquinol. FSP-1 is anchored to the plasma membrane via myristoylation, NADPH-dependent, and prevents lipid peroxidation of membrane lipids using ubiquinol as a lipophilic antioxidant [79].

Conversely, genetic conditions such as deletion of *Acsl4* or *Lpcat3*, which encode acyl-CoA synthetase long-chain family member (ACSL4) and lysophosphatidylcholine acyltransferase 3 (LPCAT3), respectively, both involved in PUFA incorporation into membranes, confer ferroptosis resistance [80,81] (Figure 1). Arachidonoyl (AA) rich phospholipids can be oxidized to produce LPO by ACLS4, producing acyl CoA derivatives. Then, LPCAT3 forms phosphatidylethanolamines, which will be substrates for lipoxygenases. In turn, as we might expect, the third mechanism that induces ferroptosis is the inhibition of lipoxygenases. Baicalein is a selective lipoxygenase inhibitor with iron-binding properties and anti-Fenton reaction bioactivity, acting as a negative regulator of ferroptosis [82].

Mitophagy and mitochondrial dynamics, mediated by fission and fusion events, regulate ferroptosis sensitivity. Disrupting this homeostasis by blocking fission using CRISPR/Cas9-mediated genetic depletion of dynamin-related protein 1 (DRP1) confers resistance to ferroptosis, whereas the lack of optic atrophy 1 type (OPA1) protects against ferroptosis through cysteine deprivation, xCT inhibition, and GPx4 inhibition [83,84]. Moreover, a protective mechanism against increased fission events is mediated by FSP1 upregulation at the cell membrane. Depleting FSP1 in these contexts restores ferroptosis susceptibility, confirming its essential role when mitochondrial dynamics are disturbed [85].

On the other hand, a mechanistic link between cardiolipin-dependent mitophagy and ferroptosis has been proposed, although it has not been directly reported. Following traumatic brain injury, cardiolipin is externalized to the mitochondrial surface via phospholipid scramblase-3 (PLS3), indicating mitochondrial damage. An early response to brain injury is the activation of the mitophagy program [86]. Therefore, a failure of the mitophagy process could lead to the accumulation of peroxidized mitochondria, enhancing neuronal death by ferroptosis, and increasing neuronal susceptibility.

Several kinds of cell death rarely occur in an isolated way. In many human diseases, ferroptosis shares signaling pathways with other forms of cell death, in which increased ROS is a common factor. ROS generated by an iron overload, the hallmark of ferroptosis, can also trigger apoptosis. For example, ferroptosis inducers such as erestin trigger ER stress in cancer cell lines, leading to increased p53 upregulated modulator of apoptosis (PUMA) gene expression via C/EBP Homologous protein (CHOP), which bridges ferroptosis and apoptosis [87]. Ferroptosis repressors, including SLC7A11 and GPX4, are degraded by the ubiquitin-proteasome system. Conversely, ferroptotic death is promoted by the overactivation of selective autophagy pathways, through which ferritin, lipid droplets, circadian proteins, and GPX4 are degraded [88]. Along the same lines, selective autophagy, which mediates the degradation of specific targets within the cell, has been linked to ferroptosis through mechanisms such as ferritinophagy, which the cell uses to release iron from ferritin via NCOA4 [89]. This process is coordinated with lipophagy through RAB7A. Key regulatory mechanisms are also identified. Beclin 1, when phosphorylated by AMPK, is demonstrated to inhibit the SLC7A11 antiporter, thereby triggering lipid peroxidation. High mobility group B1 (HMGB1) plays a dual role, being released during ferroptosis to modulate inflammatory responses as a danger signal. The contribution of autophagy to iron overload and lipid metabolism is explained; iron overload is potentiated via ferritinophagy, while lipid metabolism is affected through lipophagy, consequently enhancing the Fenton reaction and the activity of ACSL4 and ALOX enzymes, which are essential for lethal phospholipid peroxidation.

## 7. Disequilibrium of Mitochondrial Iron Handling in Diseases

### 7.1. Obesity

A nascent body of evidence suggests mitochondrial iron imbalance as part of the mechanism underlying metabolic disease. Mitoferrin 1 (MFRN1), coded by the *Slc25a37* gene, is an importer of iron in the mitochondria. In obesity-resistant mice (mice that do not develop obesity despite being fed a high-fat diet), this gene was overexpressed 4.9-fold compared to control mice. This phenotype had a better fat-to-muscle ratio, increased type 2a oxidative skeletal fibers (fast-twitch), and likely increased the capacity to oxidize fat and enhance ATP production [90]. Considering that skeletal muscle is an active tissue responsible for regulating metabolism, iron import is relevant to maintain the synthesis of mitochondrial Fe-S clusters and heme. Unlikely, overexpression of the *Slc25A28* gene, which encodes mitoferrin-2 (MFRN2), enhances diet-induced obesity in mice by increasing fat storage through increased lipogenesis and inhibition of lipolysis. Also, UCP-1 and PGC-1α are downregulated, key proteins for thermogenesis and mitochondrial biogenesis, respectively [91]. MFRN2 is part of the iron transporter SLC25 family and is located in the inner membrane of the mitochondria to import Fe^2+^ from the cytosol to the mitochondrial matrix. An alteration in *Slc25A28* expression could disrupt mitochondrial iron homeostasis, increasing mtROS production and thereby affecting the ETC and energy production in the cell [92].

### 7.2. MASLD

**Figure 3:**
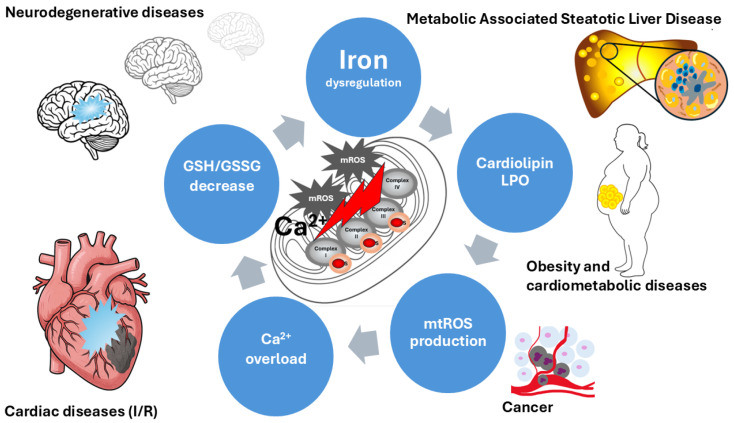
Hallmarks of mitochondrial dysfunction associated with mitochondrial iron homeostasis disturbance. The current picture shows that mitochondrial iron dysregulation is a primary trigger of cardiolipin peroxidation and increased mtROS production. Additionally, Ca^2+^ overload and a significantly decreased GSH/GSSG ratio are depicted as critical hallmarks of this pathological cycle. These interconnected dysfunctions are associated with the progression of neurodegenerative diseases, obesity, and MASLD.

Some reports suggest that iron stores are implicated in the pathogenesis of MASLD, which is consistent with the role of iron in Fenton reaction-dependent LPO. Hepatic biopsies from patients with MASLD have high hepatic iron deposition as a predictor of advanced fibrosis and histologic damage [93]. Since insulin resistance is a key pathogenic feature of MASLD, significant improvements in insulin levels and the Homeostatic Model Assessment for Insulin Resistance (HOMA-IR) index, and a higher rate of improvement in histological liver damage were reported in iron-depleted patients treated with phlebotomy [94]. Evidence from cell cultures, animal models, and humans shows that iron overload in the organism is a driving force in the MASLD progression. In the presence of hepatic inflammatory infiltrates, the addition of damage-associated molecular patterns (DAMPs) to the increase in LPO and ROS induces ferroptosis. This local environment and iron-mediated signaling can activate hepatic stellate cells (HSCs), which can differentiate into fibroblasts and produce collagen, contributing to the development of fibrosis (Figure 3) [95].

Several proteins regulate iron homeostasis; ferritin is an iron-storing protein, and elevated levels are found in many patients with NAFLD. Also, hepcidin, an intracellular iron sensor, is upregulated in obese mice [96]. In addition, transferrin receptor-1 (TfR1) is upregulated in HFD-fed mice, despite this protein being expected to be downregulated in intracellular iron overload [97]. Upregulation of TfR1 could lead to hepatocellular iron uptake in NAFLD despite increased hepatocellular iron, promoting the Fenton reaction and consequently lipoperoxidation and iron-dependent liver damage.

### 7.3. Cardiac Dysfunction

A classic model of ischemia/reperfusion (I/R) applied to cardiac tissue; mitochondrial iron imbalance is a protagonist. I/R induces an increase in intracellular iron, which triggers the vicious cycle: mtROS increase- LPO-Calcium intake, MCU activation, opening of mPTP, and cellular death. Various strategies to reduce mitochondrial iron have shown promise in reversing I/R injury. Inhibitors of ferroptosis, iron chelators, and MCU inhibitors are among the pharmacological strategies to induce cardioprotection [98].

This cascade highlights key interrelated mechanisms that are implicated in cellular damage and associated pathologies.

### 7.4. Neurodegenerative and Neuropsychiatric Disorders

The relationship between ferroptosis and the development of neurodegenerative diseases (ND) is a topic of enormous research nowadays. Iron dysregulation and LPO are also present in both the generation and progression of ND, such as Alzheimer’s (AD) and Parkinson’s diseases (PD). The last one is characterized by a loss of dopaminergic neurons in the substantia nigra and the accumulation of α-synuclein (α-syn) misfolding. Then, mtROS increases α-syn aggregation, a hallmark of the disease: Lewy bodies [99]. Increased iron, decreased total GHS levels, and mitochondrial complex I dysfunction are some of the findings reported in the substantia nigra [100,101]. It has been proposed that upregulation of heme oxygenase-1 (HO-1) in Lewy bodies, induced by neuronal stress, is responsible for the accumulation of iron inside mitochondria [102]. Aggregates of α-syn interact with mitochondria, resulting in mitochondrial membrane depolarization, decreased ATP production, mitochondrial fragmentation, and subsequent degradation via cardiolipin–dependent mitophagy [103]. Moreover, mitochondrial dysfunction increases free iron levels and LPO. Another finding is the SLC25A39 degradation, which reduces the GSH uptake, Krebs ’cycle dysregulation increases PUFA synthesis, increasing the substrate offer to ferroptosis, all of these findings promote the dopaminergic neurons’ death [101].

The pathophysiology of the disease comprises the formation of neurofibrillary tangles in neurons, caused by the abnormal aggregation of β-amyloid protein (Aβ) and the phosphorylation of tau protein, which constitute one of the main pathological features of AD [104]. Lactylation is a post-translational modification of proteins, and tau is susceptible to it. Lactylation of tau at K677 was associated with erastin-induced ferroptosis in cells treated with Aβ, opening a new field in which this tau modification could decrease iron levels and inhibit ferroptosis in microglial cells [105].

Mitochondrial dysfunction is part of the pathophysiology of AD. In a mouse model of AD, a decrease in complex I activity was found. The ATP deficit activated AMPK, triggering metabolic reprogramming and altering the membrane phospholipid composition; diacyl- and lyso-phosphatidylcholine levels increase, while ethanolamine plasmalogens and cardiolipin content decrease [106].

The role of iron in the pathophysiology of Alzheimer’s disease has become progressively less controversial, and therapeutic approaches targeting the reduction of iron levels are already being implemented. Iron dysregulation in the brains of patients with AD leads to an excess of free iron, which promotes oxidative stress and lipid peroxidation via the Fenton reaction. Ferritinophagy, a selective autophagy process mediated by nuclear receptor coactivator 4 (NCOA4) that releases iron from ferritin, is considered to act as an upstream trigger of ferroptosis in AD. Therefore, modulation of ferritinophagy and ferroptosis, particularly by targeting nuclear receptor coactivator 4, is a promising therapeutic strategy for intervening in the pathogenesis and progression of AD [107]. Conversely, maneuvers to decrease brain iron content yield contradictory results: using an iron chelator, such as deferiprone, reduced hippocampal iron accumulation in Alzheimer’s patients, but this intervention was detrimental to cognitive function [69]. These observations strongly support the hypothesis that ferroptosis is not merely an epiphenomenon but an active driver of neuronal degeneration in various neurodegenerative diseases. FSP1 acts independently of the GPX4 pathway, supporting GPX4-mediated neuroprotection, which is why FSP-1 upregulation in ferroptosis is a current research target as a potential therapeutic for neurodegenerative disorders. It has described the action of anthocyanin treatments, which upregulate FSP1 expression and restore mitochondrial integrity in neurons from a vascular dementia model [108]. While GPX4 directly reduces lipoperoxidation, its activity is modulated by the availability of its cofactor, GSH, the synthesis of which is influenced by the cysteine/glutamate antiporter system Xc−. When GPX4 is overwhelmed, FSP1 compensates. FSP1 operates independently to regenerate ubiquinol, which acts as a lipophilic radical-trapping antioxidant. Therefore, a synergistic relationship is suggested between the GSH/GPX4 axis and the FSP1/CoQ10 pathway, providing a two-layered defense where backup systems are activated to maintain neuronal redox homeostasis when primary mechanisms are inhibited. Consequently, therapeutic strategies aimed at modulating iron homeostasis, enhancing antioxidant defenses, or directly inhibiting lipid peroxidation are regarded as promising approaches. However, their clinical translation is considered to require careful evaluation of potential adverse effects.

Neuropsychiatric disorders are a broad range of entities, and depression is one of these. An interesting point of view related to the link between ferroptosis and metabolic maladaptation has been proposed as an axis present in depressive disorders [109]. Here, iron overload triggers lipid peroxidation and inactivates GPX4 and ACSL4, leading to damage to both neuronal membranes and mitochondrial cardiolipin. Mitochondrial dysfunction drives an unsustainable shift toward glycolysis, ultimately suppressing mitochondrial biogenesis by downregulating PGC-1α/TFAM pathways, as has been reported in a neuropath pain- depression model [110]. The pathological cascade is further perpetuated by the release of mitochondrial DNA, which activates microglial M1 polarization via the cyclic GMP-AMP synthase (GAS)-Stimulator of Interferon Genes (STING) pathway, thereby sustaining neuroinflammation [111]. Therapeutic strategies have been used to recover depression alterations; using a selective inhibitor of NADPH oxidase 4, ROS production was decreased and subsequently lipid peroxidation. The therapeutic effect was achieved by activating the Nrf2/HO-1/GPX4 signaling pathway, a cascade known to restore redox homeostasis and protect against iron-dependent cell death in a chronic unpredictable mild stress (CUMS) mouse model [112]. In the same model, treatment with cymaroside, a flavonoid compound, decreased microglial activation in the hippocampus, though this was mediated by modulation of the IRF1/SLC7A11/GPX4 signaling pathway [113].

### 7.5. Targeting Ferroptosis in Cancer: From Tumorigenesis to Therapy

Mitochondrial iron dysregulation has been closely linked to oncogenesis. Cancer cells exhibit an elevated demand for iron to support essential mitochondrial functions, including ETC, OXPHOS, and hemoprotein synthesis, all of which are heightened in hyperproliferative cells. Accordingly, increased iron imports are observed across various cancer cell lines, thereby promoting tumorigenesis. This enhanced iron uptake is often driven by the upregulation of specific iron-related pathways, including those involving siderophores, lipocalin 2 (LCN2) and its receptor LCN2R (SLC22A17), and cluster of differentiation 44 (CD44) [114]. Mitochondria play a critical role in hematological carcinogenesis through their involvement in Fe-S cluster biogenesis, as evidenced by disruptions in this process being linked to malignant transformation [115]. It was demonstrated that Gpx4 knockout accelerates a Kras-driven mouse model of pancreatic ductal adenocarcinoma (PDAC), and this acceleration can be reversed by liproxstatin-1 (a ferroptosis inhibitor). This finding linked ferroptosis to the pathophysiology of PDAC [116].

Multiple therapeutic targets against cancer, focusing on the stabilization of iron homeostasis. First, the stability of Fe-S clusters can be disrupted by redox manipulation: the oxidation of [4Fe-4S]^2+^ clusters by ROS generated by pharmacological ascorbate or ionizing radiation leads to enzyme inactivation and the release of redox-active iron. Iron availability for cluster formation can be limited by iron chelation, which is achieved with compounds such as deferasirox to sequester intracellular iron [117]. Finally, iron mimicry with gallium (Ga^3+^) is employed to antagonize iron-dependent processes; Ga^3+^ is taken up by cancer cells and incorporated into metabolic pathways, thereby inhibiting essential Fe-S cluster-containing enzymes, including those involved in DNA synthesis and repair [118].

Using mitochondrially targeted deferoxamine, Fe-S biogenesis is inhibited. This is a relevant potential therapeutic target because it regulates aconitase, inhibiting the TCA cycle and, in turn, OXPHOS. These events have consequences: an increase in mtROS production, which triggers a compensatory increase in glycolysis; then, mitophagy is induced, leading to the final death of cancer cells [116].

Considering the crucial role of mitochondrial iron homeostasis in sustaining tumor growth, this is a potential therapeutic approach that, by manipulating redox, chelation, and iron mimicry, can effectively inhibit cancer cell proliferation and survival.

## 8. Discussion

There has been a focus on mitochondria not just as passive recipients of oxidative damage but also as active modulators of iron-dependent redox signaling. Classically, the mitochondrion is the motor of the cell; a complex and exquisite regulation of redox, iron, and energy homeostasis is required for healthy function. Indeed, impairment of any of these processes could disrupt equilibrium, triggering a vicious cycle of damage (Figure 2). Then, the highly mitochondrial iron-handling implicated several proteins that protect cells against iron-dependent damage by the Fenton reaction. This review presents a comprehensive paradigm that identifies mitochondrial iron homeostasis and lipid peroxidation as pivotal factors in ferroptosis across diverse disease contexts. 

Although persuasive data have been compiled associating iron–sulfur cluster disruption, cardiolipin peroxidation, and the overproduction of mitochondrial reactive oxygen species with ferroptosis, certain limitations must be recognized. A significant portion of the mechanistic insights presented have been obtained from in vitro systems or animal models, which may inadequately represent disease complexity and cell-type specificity.

There have been many discussions about the therapeutic implications, especially those focusing on mitochondrial iron transport, glutathione metabolism, or lipid peroxidation. Nonetheless, altering these pathways has yielded context-specific outcomes, as iron chelation or antioxidant approaches may interfere with critical physiological processes. Consequently, the translational significance of ferroptosis-targeted therapies must be regarded with circumspection. Future research is necessary to thoroughly investigate the geographical, temporal, and tissue-specific regulation of mitochondrial iron metabolism, preferably utilizing integrated omics and sophisticated in vivo models.

The current review focused on the central role of mitochondrial iron homeostasis, raising an unanswered question, and several critical gaps remain that temper our full understanding and constrain therapeutic development. First, the precise molecular changes that trigger the shift from physiological mitochondrial redox signaling to pathological ferroptotic lipid peroxidation are still unclear. Second, what cellular types are more susceptible to mitochondrial damage mediated by cardiolipins and LPO? The interplay between ferroptosis and other forms of cell death is only beginning, and this question is crucial for identifying possible therapeutic targets in cancer.

## 9. Conclusions

Iron in mitochondria is exquisitely regulated by several proteins to prevent its toxic effects when free, as it catalyzes the production of damaging free radicals. Iron overload and oxidative stress can exacerbate its toxicity through lipid peroxidation. Ferroptosis is an iron-dependent form of cell death that, in some diseases, is triggered by lipid peroxidation (in the context of increased PUFA), increased mtROS, and iron dysregulation. The Fenton reaction occurs in this dysregulation, characterized by Fe-S cluster dysfunction and problems in heme metabolism. Locally, mtROS causes peroxidation of cardiolipin, membrane instability, Ca^2+^ overload, and mitochondrial dysfunction. Disrupted mitochondrial iron homeostasis is therefore a pivotal pathogenic pathway linking metabolic and neurodegenerative diseases.

## Data Availability

No new data were created or analyzed in this study. Data sharing is not applicable to this article.

## Abbreviations

The following abbreviations are used in this manuscript:

4-HNE	4-hydroxy-2-nonenal
AA	Arachidonoyl
ACSL4	Acyl-CoA Synthetase Long-Chain Family Member 4
ALA	5-aminolevulinic acid
ALAS1	5-aminolevulinate synthase
ATP	Adenosine triphosphate
CL	Cardiolipin
COX-10	Heme A:farnesyltransferase (Complex IV assembly cofactor)
ETC	Electron transport chain
Fe-S	Iron–sulfur
GPX4	Glutathione Peroxidase 4
GSH	Reduced glutathione
GSSG	Oxidized glutathione
HIFα	Hypoxia-inducible factor alpha
HOSS	Homeostatic Oxygen-Sensing System
LPCAT3	Lysophosphatidylcholine Acyltransferase 3
LPO	Lipoperoxidation
MASLD	Metabolically Associated Steatotic Liver Disease
MCU	Mitochondrial Calcium Uniporter
MDA	Malondialdehyde
MFRN1	Mitoferrin 1
MFRN2	Mitoferrin 2
mNCE	Mitochondrial Na^+^/Ca^2+^ Exchanger
mPTP	Mitochondrial permeability transition pore
mtROS	Mitochondrial reactive oxygen species
NADPH	Nicotinamide adenine dinucleotide phosphate
OXPHOS	Oxidative phosphorylation
PGC-1α	Peroxisome proliferator-activated receptor gamma coactivator 1-alpha
PHDs	Prolyl-hydroxylases
PUFAs	Polyunsaturated fatty acids
Q-cycle	Quinone cycle (electron transfer mechanism in Complex III)
RNS	Reactive Nitrogen Species
ROS	Reactive oxygen species
SLC25A39	Solute carrier family 25 member 39 (mitochondrial glutathione transporter)
UCP-1	Uncoupling protein 1
UCPs	Uncoupling proteins
